# Acute ayahuasca administration attenuates binge‐like eating and anxiety‐related behaviors in mice subjected to intermittent fasting

**DOI:** 10.1111/jne.70290

**Published:** 2026-10-12

**Authors:** Cristian Ferreira Corona, Felipe Alves Alencar Lima, Jessica Sabrina Canedo da Silva Sumensse, Kevin Donansan Carvalho, Pedro Henrique Grignet, Heloisa Deola Confortim, Maria Claudia Gross, Fabiana Aidar Firmino, Fernanda Ferreira Evangelista, Francisney Pinto Nascimento, Jean Franciesco Vettorazzi

**Affiliations:** ^1^ Latin American Institute of Life and Nature Sciences (ILACVN) Federal University of Latin American Integration (UNILA) Foz do Iguaçu Paraná Brazil; ^2^ Federal University of Paraná—Toledo Toledo Paraná Brazil

**Keywords:** Ayahuasca, binge‐like eating, CART, hypothalamus, intermittent fasting

## Abstract

Human binge eating disorder is characterized by recurrent episodes of excessive food consumption accompanied by loss of control and is strongly associated with alterations in reward processing and emotional regulation. Ayahuasca, a psychoactive botanical preparation traditionally used in Amazonian medicine, has been shown to modulate serotonergic signaling and reward‐related behaviors and has emerged as a potential therapeutic tool for mood and substance use disorders. However, its effects on binge‐like eating behavior remain largely unexplored. In the present study, we evaluated the behavioral and metabolic consequences of a binge‐like eating model in mice and investigated the effects of a single administration of ayahuasca on food intake, sucrose consumption, and anxiety‐like behavior. Male C57BL/6 mice were subjected to an intermittent fasting protocol combined with restricted access to a standard diet and a sucrose solution for 10 days. On the eleventh day, a subgroup of binge‐exposed mice received a single oral dose of ayahuasca (1.76 mg/kg of N, N‐dimethyltryptamine). Food and sucrose intake were quantified during refeeding periods; anxiety‐like behavior was assessed using the elevated plus maze; and the expression of hypothalamic genes POMC and CART was evaluated by RT‐PCR. Mice exposed to the binge‐like eating protocol exhibited increased food and sucrose intake during restricted access periods, reduced body weight, anxiogenic‐like behavior, and reduced hypothalamic CART gene expression. Ayahuasca administration selectively reduced food and sucrose intake during the initial hour of refeeding without affecting later intake, reversed anxiety‐like behavior, and increased hypothalamic CART expression. These findings support the notion that ayahuasca acutely attenuates binge‐like eating and anxiety‐related behaviors in mice, supporting its potential relevance for investigating psychedelic‐based approaches targeting compulsive eating and associated emotional dysregulation.

## 1 INTRODUCTION

Binge eating disorder in humans is characterized by recurrent episodes of binge eating occurring within a short period of time, involving the consumption of large amounts of food, accompanied by a loss of control over eating. The worldwide prevalence of binge eating disorder is estimated to be approximately 0.6–1.8% among adult women and 0.3–0.7% among adult men between 2018 and 2020, and this condition is strongly associated with obesity and mental health alterations.^1^

Binge eating disorder was formally included in the Diagnostic and Statistical Manual of Mental Disorders (DSM) in 2013^2^ and in the International Classification of Diseases (ICD) in 2019.^3^ In humans, the disorder is defined by the ingestion of unusually large amounts of food within a discrete period, typically around 2 h, occurring at least once per week for a minimum of 3 months and accompanied by marked distress related to binge eating episodes.^4^ Increased consumption of sugar‐sweetened beverages and binge‐like patterns of sugar intake have been associated with a broad spectrum of health and behavioral problems, including affective disorders, substance abuse, obesity, diabetes, and metabolic syndrome.^5^

Although only recently recognized as a distinct clinical syndrome, binge eating disorder is the most prevalent eating disorder. This condition is frequently accompanied by psychiatric comorbidities, including mood disorders, anxiety disorders, and substance use disorders.^6^ Conversely, eating disorder symptoms have been associated with subsequent anxiety symptoms, longitudinal increases in anxiety severity, and a higher risk of developing anxiety disorders.^7^ Alterations in reward sensitivity and food‐related attentional biases are hallmark features of binge eating disorder, involving dysfunctions in corticostriatal circuits that closely resemble those observed in substance abuse. Accordingly, binge eating disorders share characteristics related to impaired impulse control and motivation with impulsive and compulsive disorders.^8^

The main mechanisms proposed for binge eating are related to mesolimbic dopaminergic dysfunctions, but the hypothalamus also plays a significant role in this behavior.^9^, ^10^ Metabolic signals can enhance or attenuate the hedonic value of food. In this context, the lateral hypothalamus (LH) acts as a major relay center between metabolic and reward‐related circuits.^11^, ^12^, ^13^ This region receives projections from orexigenic neurons in the arcuate nucleus (ARC) that express neuropeptide Y (NPY) and agouti‐related peptide (AgRP).^14^, ^15^ Upon activation by ghrelin during fasting, these neurons inhibit the paraventricular hypothalamus (PVH) and increase orexinergic signaling within the LH, thereby enhancing food‐seeking behavior and facilitating dopaminergic activity in the ventral tegmental area (VTA). Conversely, anorexigenic pro‐opiomelanocortin/cocaine‐ and amphetamine‐regulated transcript (POMC/CART) neurons in the ARC are activated by metabolic satiety signals such as leptin and insulin, increasing melanocortin signaling in the PVH while suppressing NPY/AgRP neuronal activity.^16^, ^17^ In this framework, leptin and ghrelin exert opposing effects on feeding and binge‐like eating by differentially recruiting distinct neuronal populations, linking peripheral metabolic status to hedonic eating.^18^, ^19^

It is established the anorexigenic effect of serotonergic agonist administration in the hypothalamus.^20^, ^21^, ^22^ This effect is mediated by the expression of 5‐HT_2C_ receptors on POMC/CART neurons, as well as inhibitory presynaptic 5‐HT_1B_ receptors on NPY/AgRP neurons.^23^ Moreover, the presence of 5‐HT_2A_ receptors on POMC/CART neurons^24^ provides a rationale for investigating the potential use of psychedelics, botanical complexes known for their direct, albeit not exclusive, action on 5‐HT_2A_ receptors. Although direct evidence linking ayahuasca to hypothalamic POMC/CART regulation remains limited, serotonergic psychedelics exert their effects primarily through 5‐HT2A receptor activation and have been shown to influence feeding behavior, reward processing, and motivational states, supporting the investigation of their potential effects on hypothalamic circuits involved in energy balance.

Besides the recently approved lisdexamfetamine as a treatment for binge eating disorders,^25^ current treatment strategies are primarily based on cognitive‐behavioral therapy, dialectical behavior therapy, and motivational interviewing.^26^ In this context, substances capable of modulating reward processing, compulsive behavior, and addiction‐related pathways represent promising therapeutic alternatives. Ayahuasca is a psychedelic brew traditionally used in the Amazon rainforest, composed of the *Banisteriopsis caapi vine* and a plant containing the potent psychedelic compound dimethyltryptamine (DMT), most commonly *Psychotria viridis*.^27^

Both animal and human studies have demonstrated that ayahuasca exerts anxiolytic and antidepressant effects.^28^ Ayahuasca and DMT per se modulate the immune system and reduce pro‐inflammatory cytokine levels associated with depressive‐like states in rats.^29^ Furthermore, ayahuasca and its alkaloids, such as harmine, tetrahydroharmine (THH), and harmaline, reduce cocaine and morphine consumption in rats,^30^ modulate dopaminergic signaling in the nucleus accumbens,^31^ and attenuate ethanol‐induced behavioral sensitization in mice by reducing acute sensitized responses.^32^ In humans, evidence indicates that ayahuasca reduces alcohol consumption in adolescents^33^ and cocaine abuse,^34^ while also improving personality traits, psychopathology, cognition, life attitudes, and spirituality.^35^ To date, experimental evidence regarding the effects of ayahuasca on binge‐like eating behavior remains scarce.

In summary, although the therapeutic effects of Ayahuasca have been largely attributed to molecular changes in cortical networks,^30^, ^31^, ^32^, ^33^, ^34^, ^35^ the hypothalamus remains poorly explored in this context. Therefore, we hypothesized that Ayahuasca administration could attenuate binge‐like eating behavior and modulate the expression of anorexigenic hypothalamic genes associated with energy balance regulation. Accordingly, the present study aimed to evaluate the effects of binge‐like eating on food intake and behavioral outcomes in mice, as well as to investigate the potential therapeutic effects of Ayahuasca on these behaviors and the associated hypothalamic gene expression patterns.

## 2 MATERIALS AND METHODS

### 2.1 Ethics and animals

All experiments involving animals were authorized by the Ethics Committee for the Use of Animals (CEUA, protocol no. 23422.013429/2023–04) of the Federal University of Latin American Integration (UNILA). Male *Mus musculus* mice of the C57BL6 strain weighing 20–25 g were used, aged 30 days. Animals were housed in a climate‐controlled animal room (21 ± 2°C; 60% relative humidity) under a 12‐h light/dark cycle (lights on at 7:00 AM and off at 7:00 PM). Before the experimental protocol, animals were acclimatized for 2 weeks and had free access to water and food.

### 2.2 Experimental model

After acclimatization in groups of four or five animals per cage, the animals were divided into two groups: the control group (CTL, *n* = 8), which received a standard diet (AIN93—Rhoster Indústria e Pesquisa, São Paulo, Brazil), and the binge‐like eating group (BLE, *n* = 16). After the 10‐day binge‐like eating protocol, the BLE group was subdivided into BLE (*n* = 8) and BLE + AYA (*n* = 8). Each animal's tail was marked with a nontoxic marker, which was reapplied when necessary, allowing individual animals to be followed throughout the study.

To induce a murine model of binge‐like eating, the animals were subjected to an intermittent fasting protocol consisting of two stages repeated over 10 consecutive days: (i) animals were maintained in their group‐housed cages under food restriction for 20 h, from 1:00 PM to 9:00 AM; followed by (ii) a 4‐h period in individual cages, from 9:00 AM to 1:00 PM, during which they had free access to standard chow and a 0.5 M sucrose solution. After this 4‐h period, animals were returned to their group‐housed cages. Thus, individual housing was restricted to the 4‐h daily intake‐measurement period throughout the 10‐day protocol. Water was available ad libitum throughout the experimental period.

The CTL group was not subjected to food restriction or sucrose exposure. However, CTL animals underwent the same handling and housing procedures, including transfer to individual cages during the corresponding 4‐h period, to ensure an equivalent level of manipulation. Body weight and food and water (or sucrose) intake were recorded daily at a consistent time, coinciding with the transfer of the animals to their individual cages.

On day 11, sucrose was withheld to induce withdrawal. After confirmation of the BLE phenotype, BLE mice (*n* = 16) were subdivided into two treatment subgroups and received a single oral gavage of either ayahuasca at a dose of 1.76 mg/kg DMT (BLE + ayahuasca; *n* = 8) or saline (BLE + Saline; *n* = 8), while control mice received saline (CTL + Saline; *n* = 8). Thirty minutes post‐treatment, food and water intake were measured for 4 h^36^ in individual cages, without sucrose.

On day 12 (48 h without sucrose), anxiety‐like behavior was assessed via the Elevated Plus Maze (EPM) during the animals' usual sucrose‐access window (9:00 AM–1:00 PM). At 1:00 PM, BLE mice were re‐exposed to chow and sucrose, with intake measured at 1 and 4 h. Following maintenance of the diet protocol through days 13–14, mice were euthanized on day 15 for hypothalamic gene expression analysis (POMC: *n* = 6/group; CART: *n* = 5/group).

### 2.3 Food intake

During all experimental periods, chow and sucrose consumption were monitored. In the BLE and BLE + AYA groups, food and sucrose intake were assessed at 1 and 4 h after the onset of the daily refeeding period. At each time point, consumption was calculated cumulatively as the difference between the amount initially offered and the amount remaining in the cage. Therefore, values recorded at 1 and 4 h represent cumulative intake over the first 1 and 4 h of refeeding, respectively, rather than intake during isolated intervals. The CTL group had chow intake measured at the same circadian time points to minimize handling‐related bias.

### 2.4 LC–MS/MS analysis and data acquisition

To quantify the concentration of ayahuasca active alkaloids (DMT, harmine, harmaline, and tetrahydroharmine) and ensure an accurate, standardized, and reproducible pharmacological dosing for the in vivo experiments, all samples underwent analysis using an LCMS‐8045 liquid chromatography‐triple quadrupole mass spectrometer (Shimadzu, Kyoto, Japan), following Eller et al.^38^ Sample analyses were performed using a liquid chromatography system coupled to a triple quadrupole mass spectrometer (LCMS‐8045, Shimadzu, Kyoto, Japan), as previously described.^38^ The mass spectrometer was operated under the following conditions: heat block temperature, 400°C; capillary voltage, 1.5 kV; nebulizing gas (N_2_) flow rate, 3 L/min; desolvation line temperature, 250°C; drying gas (N_2_) flow rate, 10 L/min; and collision‐induced dissociation gas (Ar) pressure, 230 kPa. Quantitative analysis was conducted in multiple reaction monitoring (MRM) mode, with two transitions monitored for each compound: DMT (m/z 189.0 → 58.0/144.1). Chromatographic separation was achieved using an Acquity UPLC® C18 column (2.1 × 50 mm, 1.7 μm particle size; Waters Corporation, Ireland) maintained at 60°C, with a flow rate of 0.3 mL/min. The mobile phase consisted of ultra‐pure water (A) and acetonitrile (B), both containing 0.1% (v/v) formic acid. The gradient elution program was as follows: 0–2.0 min, 3–40% B; 2.0–3.0 min, 40–97% B; and 4.5–8.0 min, re‐equilibration at 3% B. Data acquisition and processing were performed using LabSolutions software (Shimadzu, Kyoto, Japan).

### 2.5 Behavioral analysis in the elevated plus maze

The elevated plus maze (EPM) test was employed to evaluate the anxiogenic‐like effect of binge‐like eating protocol. The apparatus consisted of a plus‐sign apparatus that was 60 cm above the floor. This plus‐sign configuration consisted of two open arms across from each other and perpendicular to two closed arms, each measuring 36 cm in length and 5 cm in width. The closed arms were surrounded by 15 cm‐high wooden walls, while the open arms had a 0.5 cm lateral acrylic edge to prevent animals from falling. The behavior testing room in which the elevated plus maze was located is soundproof and low light. Testing was conducted during the light phase of the cycle, between 9:00 AM and 1:00 PM, alternating between animals from each group throughout the period to avoid time‐of‐day bias.

The maze was cleaned with a 70% alcohol solution in the interval between each animal. Behavior was recorded using a video camera [HD‐Webcam Full HD Logitech Brio 1001080p/30fps (1920 × 1080)] for a 5‐min period. Videos were analyzed using the ANY‐Maze behavioral tracking software (Stoelting Co., version 7.61, Wood Dale, IL). Behavioral measures analyzed included the number of entries and the time spent in the open arms and entries in the closed arms. The analyzed behavioral measures included: (i) the percentage of time spent in the open arms, serving as the primary parameter for anxiety‐like behavior, where a reduction relative to the control group indicates an anxiogenic state; (ii) the time spent in the central zone reflects decision‐making processes and approach‐avoidance conflict; (iii) the total distance traveled, utilized as an index of baseline locomotor activity; and (iv) the number of closed arm entries, serving as an anxiety‐independent control parameter for locomotion.

### 2.6 Real‐time PCR

At the end of the experimental period, mice were euthanized and the hypothalamic area was carefully dissected from the central nervous system. Tissue samples were immediately stored at −80°C until further processing. Total RNA was extracted using the automated Extracta® 96 system (Loccus, São Paulo, Brazil) and the Extracta Kit—MTTD‐P016 (Loccus, São Paulo, Brazil), according to the manufacturer's instructions. RNA concentration and purity were assessed prior to cDNA synthesis. cDNA was synthesized using the cDNA Synthesis Kit with RNase Inhibitor (PRT‐110 L, Cellco, São Paulo, Brazil) from 0.05 μg of total RNA. Quantitative real‐time PCR (qPCR) was performed using the qPCR Green Master Low ROX 2× kit (PCK‐101 L, Cellco, São Paulo, Brazil) in a CFX96 Real‐Time PCR Detection System (Bio‐Rad, Hercules, CA, USA). Reactions were carried out in a final volume of 20 μL containing 10 μL of qPCR Green Master Low ROX 2× (PCK‐101 L—Cellco, São Paulo—Brazil), 400 nM of forward and reverse primers, PCR‐grade water, and cDNA template. The amplification protocol consisted of initial denaturation at 95°C for 2 min, followed by 40 cycles of 95°C for 15 s and 60°C for 1 min. Melt curve analysis was performed at the end of each run to confirm amplification specificity. Melt curve analysis demonstrated a single peak for each primer pair, indicating absence of nonspecific amplification and primer‐dimer formation. All samples were analyzed in technical duplicates. Relative gene expression was calculated using the 2^−^ΔΔCt method and normalized to GAPDH expression, which was used as the endogenous housekeeping gene due to the relatively stable Ct values observed across experimental groups. Primers sequence are listed below:

**Table:** 

	Forward sequence	Reverse sequence
POMC (Proopiomelanocortin)	CGACAGGCAGGAGACTGAAC	AGATGCGCAGAGAAACGAGG
CART (Cocaine‐ and amphetamine‐regulated transcript)	ACCTTTGCTGGGTGCCCGTG	TGCAACGCTTCGATCAGCTCC
GAPDH	CCTGCACCACCAACTGCTTA	GCCCCACGGCCATCACGCCA

### 2.7 Declaration of generative AI and AI‐assisted technologies

During the preparation of this manuscript, the authors used ChatGPT (OpenAI) solely for English language editing, as English is not their native language. The authors subsequently reviewed and edited the content and took full responsibility for the accuracy, originality, and integrity of the published work.

### 2.8 Statistical analysis

Statistical analyses were performed using GraphPad Prism (version 8.0.2). Data were tested for normality (Shapiro–Wilk test) and homogeneity of variances (Levene's test). When parametric assumptions were met, Student's *t* tests (paired or unpaired) were used for single comparisons. For longitudinal datasets obtained from the same animals over time, repeated‐measures ANOVA models were employed. One‐way repeated‐measures ANOVA was used when only one factor was present (e.g., sucrose intake across acquisition days within the BLE group), whereas two‐way repeated‐measures ANOVA was applied when both time and experimental group/treatment were analyzed simultaneously (e.g., body weight and chow intake trajectories). Bonferroni post hoc tests were performed when appropriate.

For endpoint analyses, one‐way ANOVA or two‐way ANOVA was used according to the experimental design. When the assumption of sphericity was violated, the Greenhouse–Geisser correction was applied. In the presence of marked deviations from normality or outliers that could not be corrected by transformation, nonparametric tests were employed (Mann–Whitney *U* test or Kruskal–Wallis test followed by Dunn's post hoc test). Data are presented as mean ± SEM or median and interquartile range. Statistical significance was set at *p* < 0.05.

To estimate the magnitude of differences between groups, standardized effect sizes (Hedges' *g*) with 95% confidence intervals (CIs) were calculated and interpreted as negligible (*g* < 0.20), small (0.20 ≤ *g* < 0.50), moderate (0.50 ≤ *g* < 0.80), large (0.80 ≤ *g* < 1.20), or very large (*g* ≥ 1.20).

## 3 RESULTS

### 3.1 Characterization of the binge‐like eating model

Model confirmation was performed during the 10‐day acquisition period, which body weight trajectories were analyzed using a two‐way repeated‐measures ANOVA. There was a significant main effect of group (*F*(1, 22) = 33.13, *p* < 0.0001), a significant main effect of time (*F*(1,58, 34.84) = 19.26, *p* < 0.0001), and a significant time × group interaction (*F*(10,220) = 21.43, *p* < 0.0001; Figure 1A and Table S1). Post hoc comparisons indicated that BLE mice did not differ from controls on day 1 but exhibited significantly lower body weight from day 2 onwards (*p* < 0.05). BLE protocol also induced a reduction in final body weight when compared to CTL (BLE: 18.95 ± 0.38 g vs. CTL: 24.51 ± 0.83 g; unpaired *t* test), corresponding to a large, standardized effect (Hedges' *g* = 2.81; 95% CI: 1.62–4.00; Figure 1B).

**FIGURE 1:**
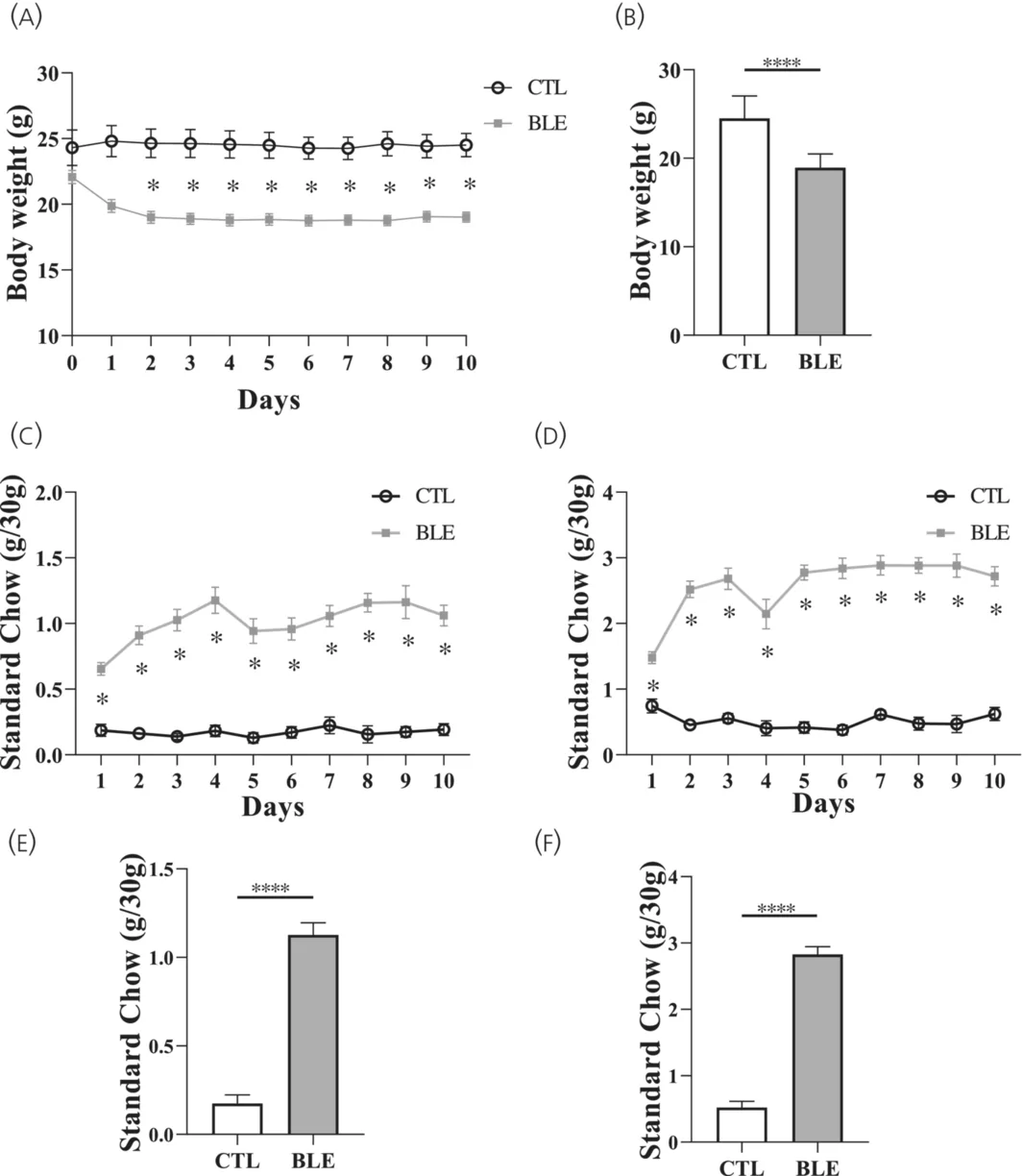
Characterization of the binge‐like eating (BLE) model. Daily body weight trajectories during the 10‐day intermittent fasting–refeeding protocol. (A). Final body weight (B). Standard chow intake during the first hour (C) and over 4 h (D) of daily access to food. Mean chow intake across the last 3 days (average of days 8–10) during the first hour (E) and over 4 h (F) of access. Data are expressed as mean ± SEM; *n* = 8 in the CTL group; *n* = 16 in the BLE group. Symbols indicate significant differences between CTL and BLE groups according to Bonferroni post hoc test.

BLE mice exhibited consistently higher standard chow intake than CTL animals during both the first hour (Figure 1C) and the cumulative 4 h period (Figure 1D) of food access. Analysis of the 1 h intake using two‐way repeated‐measures ANOVA revealed a significant main effect of group (*F*(1, 22) = 144.4, *p* < 0.0001), no significant main effect of time (*F*(4.82,101.0) = 2.11, *p* = 0.076), and a significant group × time interaction (*F*(9,198) = 2.05, *p* = 0.038; Table S2A). Bonferroni post hoc analysis demonstrated that BLE mice consumed significantly more standard chow than CTL mice at every experimental day (all *p* < 0.0001; Table S2B). This difference was particularly pronounced during the last 3 days of the acquisition period (days 8–10), with an extremely large, standardized effect size (Hedges' *g* = 3.84, 95% CI: 2.42–5.25; Figure 1E).

A similar pattern was observed for cumulative chow intake over the 4 h feeding period (Figure 1D). Two‐way repeated‐measures ANOVA revealed significant main effects of group (*F*(1, 22) = 276.4, *p* < 0.0001) and time (*F*(3,83,84.23) = 4.20, *p* = 0.0043), together with a significant group × time interaction (*F*(9,198) = 6.68, *p* < 0.0001; Table S3A). Bonferroni post hoc comparisons confirmed that BLE mice consumed significantly more chow diet than CTL animals on every experimental day (all *p* ≤ 0.0006; Table S3B). Consistent with these findings, the average chow intake during the last three acquisition days was markedly greater in BLE mice than in CTL mice, corresponding to an extremely large, standardized effect (Hedges' *g* = 5.31, 95% CI: 3.52–7.09; Figure 1F).

Together with changes in body weight and standard chow intake, BLE mice also exhibited a progressive increase in sucrose consumption during both the 1 and 4 h access periods throughout the acquisition phase (Figure 2A). One‐way repeated‐measures ANOVA revealed a significant effect of time for sucrose intake measured after 1 h (*F*(3,05, 45.80) = 34.80, *p* < 0.0001) and after 4 h (*F*(4,081, 61.21) = 114.0, *p* < 0.0001). Bonferroni post hoc analysis showed that sucrose intake was significantly higher from day 2 onward compared with day 1, with consumption progressively increasing before reaching a plateau during the final days of the protocol (Tables S4 and S5). The effect size shows that values were markedly higher at 4 h compared with 1 h (mean difference = 5.38), with an extremely large, standardized effect size (Hedges' *g* = 6.26, 95% CI: 4.53–7.99), indicating a pronounced separation between time points.

**FIGURE 2:**
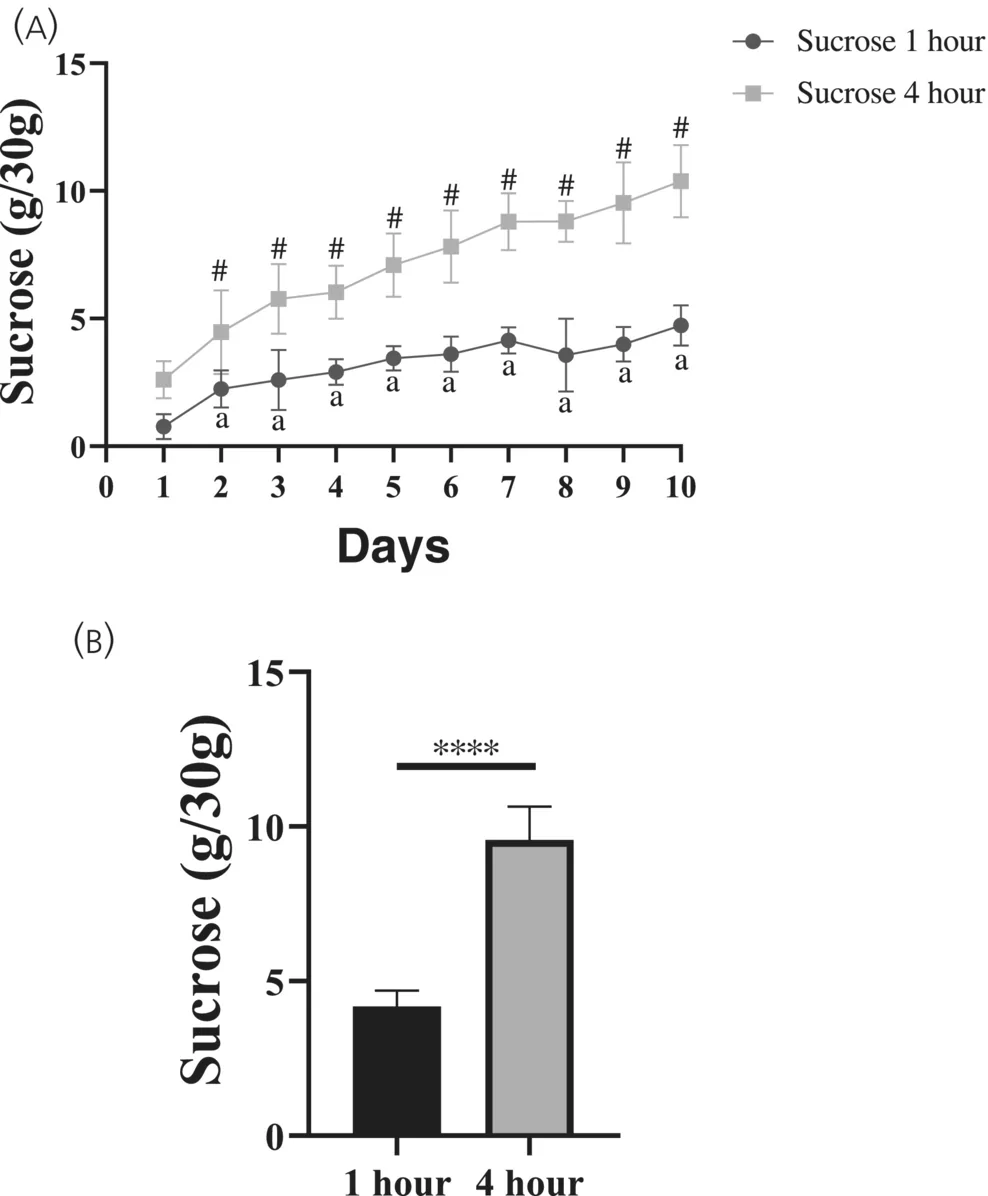
Sucrose intake during acquisition of the binge‐like eating (BLE) model. Daily sucrose intake measured after 1 h and cumulatively after 4 h of sucrose access throughout the 10‐day intermittent fasting–refeeding protocol (A). Mean sucrose intake measured after 1 h and cumulatively after 4 h across the acquisition period (B). Data are expressed as mean ± SEM (*n* = 16). a *p* < 0.05 vs. day 1 for 1‐h intake; ^#^ *p* < 0.05 vs. day 1 for cumulative 4‐h intake; ****p < 0.0001 for the comparison between 1‐h and cumulative 4‐h intake.

### 3.2 Effects of a single dose of ayahuasca on food and sucrose intake in mice submitted to binge‐like eating protocol

After the 10‐day acquisition period, BLE mice received a single oral gavage of either ayahuasca (BLE + AYA; *n* = 8) or saline (BLE; *n* = 8) on day 11. Analysis of 1 h chow intake (Figure 3A) using two‐way repeated‐measures ANOVA revealed a significant group × time interaction (*F*(4, 42) = 52.68, *p* < 0.0001), together with significant main effects of time (*F*(1,93, 40.56) = 18.09, *p* < 0.0001) and group (*F*(2, 21) = 46.68, *p* < 0.0001). Bonferroni post hoc analysis demonstrated that CTL and BLE mice maintained stable 1 h chow intake throughout the experimental period (all *p* > 0.05; Table S6), whereas BLE + AYA mice exhibited a marked reduction in intake on day 11 compared with baseline (*p* < 0.0001), followed by partial recovery on day 12 (baseline vs. day 12, *p* = 0.0676; day 11 vs. day 12, *p* = 0.0138). These findings indicate that ayahuasca produced an acute and transient suppression of chow intake, with an extremely large, standardized effect size (Hedges' *g* = 4.02, 95% CI: 2.24–5.80), an effect that was not observed in saline‐treated BLE mice.

**FIGURE 3:**
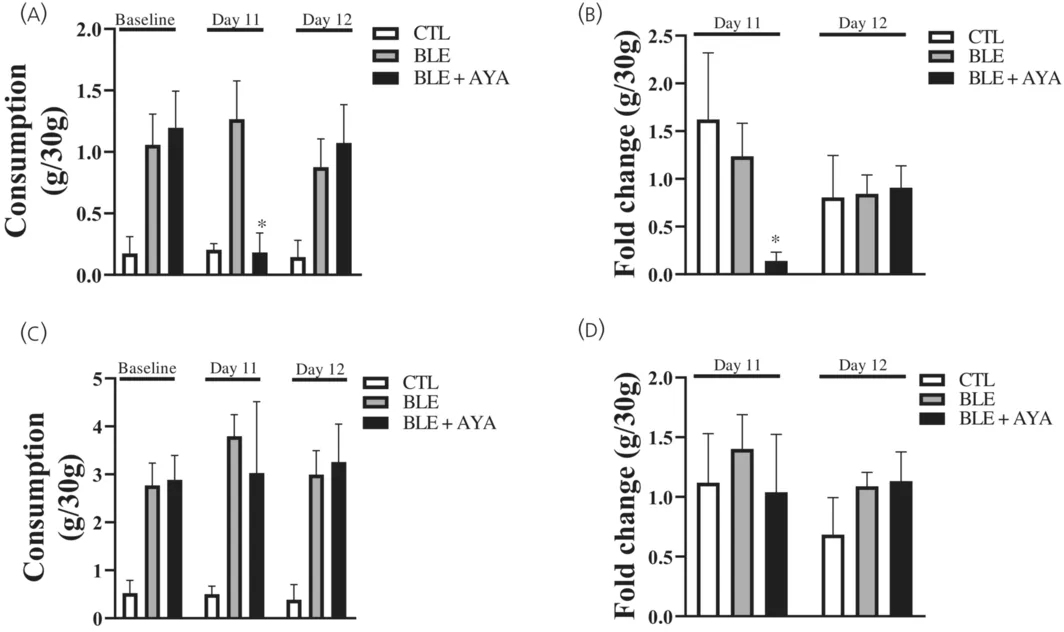
Effect of ayahuasca on standard chow intake in the binge‐like eating (BLE) model. Absolute standard chow intake during the first hour (A) and over 4 h (C) of access at baseline and on day 12 in BLE and BLE + AYA mice. Individual variation (fold change) in standard chow intake during the first hour (B) and cumulative 4 h (D) on day 12 relative to baseline. Data are expressed as mean ± SEM (*n* = 8 per group for CTL, BLE and BLE + AYA). *p < 0.05 versus the respective baseline within the same group.

A similar pattern was observed for cumulative 4 h chow intake (Figure 3C). Two‐way repeated‐measures ANOVA revealed a significant group × time interaction (*F*(4, 42) = 3.415, *p* = 0.0166) and a significant main effect of group (*F*(2, 21) = 69.38, *p* < 0.0001), whereas the main effect of time was not significant (*F*(1.38, 28.89) = 3.451, *p* = 0.0611). Bonferroni post hoc analysis showed no significant changes over time in CTL or BLE + AYA mice.

To account for interindividual variability, chow intake was also expressed as fold change relative to each animal's baseline (Figure 3B,D). On day 11, fold change in 1 h chow intake differed significantly among groups (Kruskal–Wallis: *H* = 16.21, df = 2, *p* = 0.0003). BLE + AYA mice exhibited a marked reduction relative to baseline compared with both CTL (adjusted *p* = 0.0003; Hedges' *g* = 2.81, 95% CI: 1.38–4.24) and BLE mice (adjusted *p* = 0.0100; Hedges' *g* = 4.10, 95% CI: 2.30–5.91), whereas CTL and BLE mice did not differ (adjusted *p* > 0.99; Hedges' *g* = 0.66, 95% CI: −0.35 to 1.67). Fold change in cumulative 4 h chow intake on day 11 did not differ between groups (Kruskal–Wallis: H^2^ = 3.04, *p* = 0.2193). By day 12, fold change in 1 and 4 h chow intake no longer differed among groups (one‐way ANOVA: F(2, 21) = 0.2246, *p* = 0.8007), indicating recovery from the acute anorexigenic effect of ayahuasca (Figure 3B).

Ayahuasca also altered sucrose intake following treatment (Figure 4). Analysis of 1 h sucrose intake using two‐way repeated‐measures ANOVA revealed a significant group × time interaction (*F*(1, 14) = 7.210, *p* = 0.0178) and a significant main effect of time (*F*(1, 14) = 6.406, *p* = 0.0240), whereas the main effect of group was not significant (*F*(1, 14) = 2.850, *p* = 0.1135) (Figure 4A and Table S7A). Bonferroni‐corrected post hoc comparisons revealed that BLE mice significantly increased 1 h sucrose intake from baseline to day 12 (*p* = 0.0049), corresponding to a large standardized effect (Hedges' *g* = 1.33, 95% CI: 0.40–2.26). In contrast, BLE + AYA mice showed no significant change over time (adjusted *p* > 0.9999; Hedges' *g* = −0.05, 95% CI: −0.86 to 0.76), indicating that ayahuasca prevented the increase in early sucrose intake observed in untreated BLE animals (Table S8A).

**FIGURE 4:**
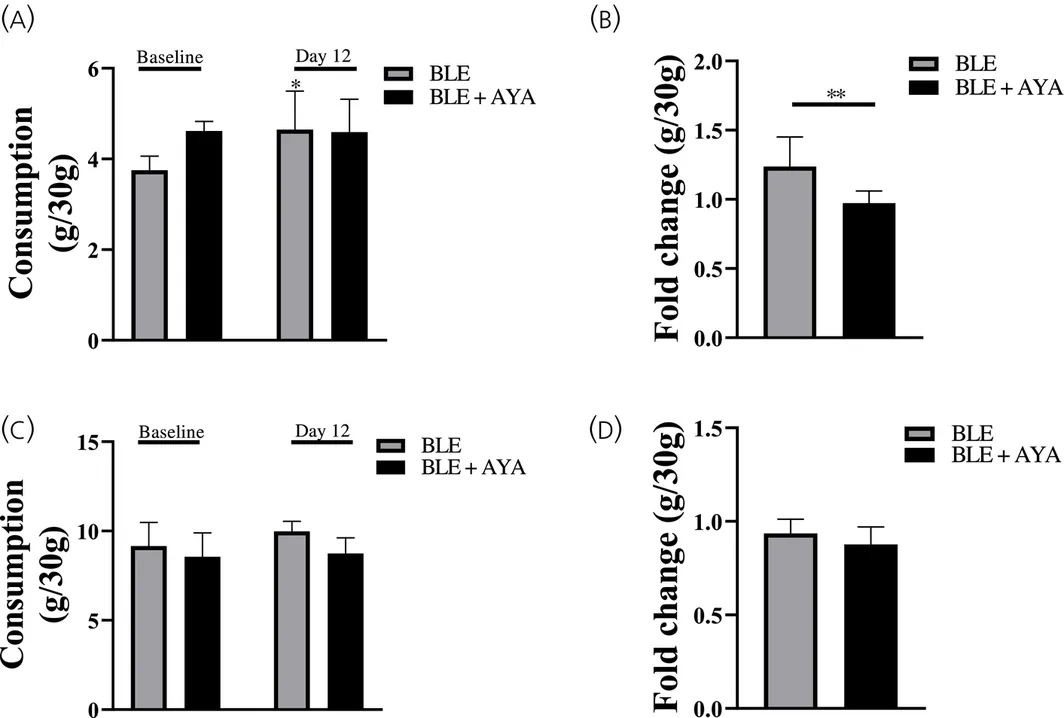
Effect of acute ayahuasca administration on sucrose intake in mice with binge‐like eating (BLE). Following a single oral administration of ayahuasca on day 11, absolute sucrose intake was measured after 1 h (A) and cumulatively after 4 h (C) at baseline (day 10) and on day 12 in BLE and BLE + AYA mice. Fold change relative to baseline is shown for 1 h (B) and cumulative 4 h (D) sucrose intake on day 12. Data are expressed as mean ± SEM (*n* = 8 per group). *p < 0.05 versus the respective baseline within the same group; **p < 0.01 between groups.

For cumulative 4 h sucrose intake (Figure 4C), two‐way repeated‐measures ANOVA revealed no significant group × time interaction (*F*(1, 14) = 0.769, *p* = 0.3954) and no significant main effect of time (*F*(1, 14) = 1.925, *p* = 0.1870), whereas a significant main effect of group was detected (*F*(1, 14) = 5.522, *p* = 0.0340) (Table S7B). However, Bonferroni‐corrected post hoc comparisons showed no significant differences between baseline and day 12 within either group (BLE: adjusted *p* = 0.2633; BLE + AYA: adjusted *p* > 0.9999), indicating that the overall group effect was not driven by treatment‐related changes over time (Table S8B).

Fold‐change analysis supported these findings. At 1 h (Figure 4B), sucrose intake differed significantly between BLE and BLE + AYA mice (Mann–Whitney U = 2.00, *p* = 0.0006). BLE mice exhibited a positive fold change relative to baseline, whereas BLE + AYA mice showed a slight reduction, corresponding to a very large between‐group effect size (Hedges' *g* = −1.57, 95% CI: −2.56 to −0.58). In contrast, fold change in cumulative 4 h sucrose intake (Figure 4D) did not differ significantly between groups (unpaired *t* test: *t*(14) = 1.365, *p* = 0.1938; 95% CI for the mean difference: −0.1489 to 0.3309), although the estimated effect size was moderate (Hedges' *g* = −0.65, 95% CI: −1.54 to 0.24).

Taken together, these findings indicate that acute ayahuasca administration prevented the increase in early (1 h) sucrose intake observed in saline‐treated BLE mice, while exerting little influence on cumulative 4 h sucrose consumption.

### 3.3 Effects of binge‐like eating model and ayahuasca treatment animal behavior

For the anxiety‐like behavior we utilized the elevated plus maze (EPM), which revealed a significant effect of the experimental condition on the percentage of time spent in the open arms [F (2. 23) = 3.60; *p* < 0.05]. BLE animals spent significantly less time in the open arms compared to the CTL animals (*p* < 0.05), with a significant effect size (Hedges' *g* = 1.42, 95% CI: 0.34–2.50), indicating an anxiogenic‐like behavior (Figure 5A). Of note, ayahuasca single dose treatment partially attenuated this BLE‐induced time in the open arms reduction, as indicated by a moderate effect size relative to BLE (Hedges' *g* = 0.76, 95% CI: −0.20 to 1.72), although the confidence interval overlapped zero. No differences were observed between CTL and BLE + AYA groups (Hedges' *g* = 0.31, 95% CI: −0.65 to 1.27).

**FIGURE 5:**
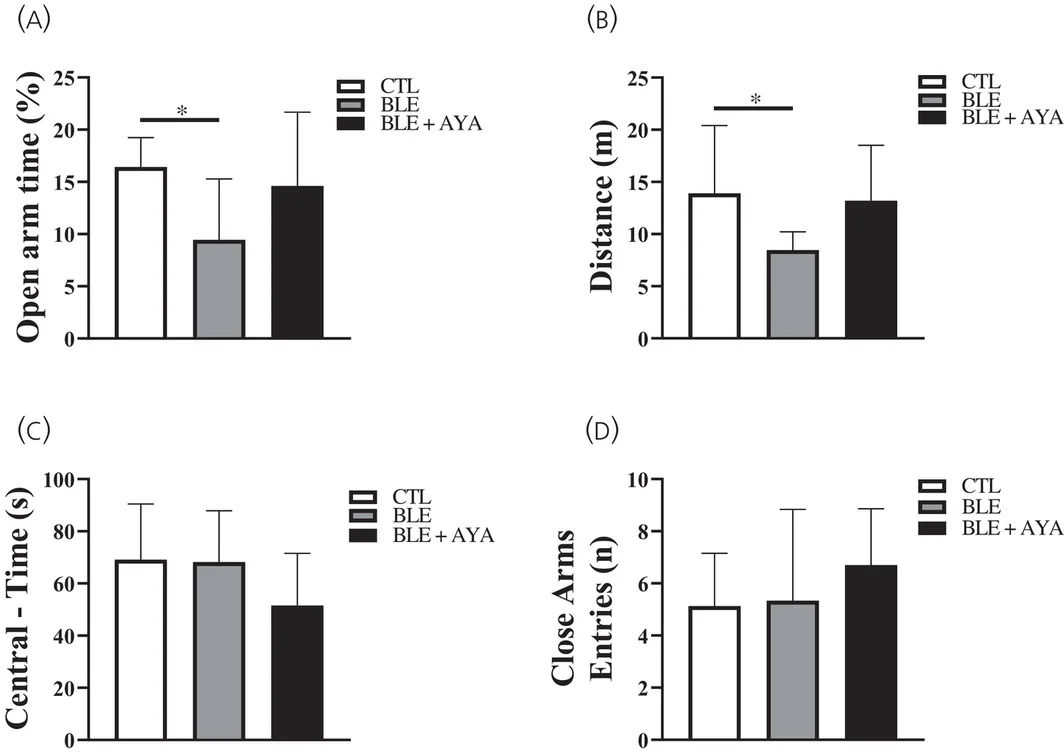
Effects of binge‐like eating (BLE) protocol and ayahuasca (AYA) treatment on anxiety‐like behavior in the elevated plus maze (EPM). Time spent in the open arms (A), total distance traveled (B), time spent in the central area (C), and number of entries into the closed arms for CTL, BLE + saline, and BLE + AYA groups (D). Data are expressed as mean ± SEM. *p ≤ 0.05; *n* = 8 per group. %: Percentage; m: Meters; s: Seconds; n: Number of entries.

Furthermore, EPM total distance traveled analysis also indicated a significant experimental effect [F (2. 23) = 4.47; *p* < 0.05]. A significant reduction in total distance traveled by the BLE group compared to the CTL group was found following post‐hoc comparisons (*p* < 0.05), whereas ayahuasca treatment reversed the BLE‐induced altered locomotor activity (Figure 5B). BLE mice exhibited lower distance traveled values in comparison to CTL mice, with a very large effect size (Hedges' *g* = 1.22, 95% CI: 0.24–2.20). Ayahuasca single dose treatment robustly reversed this BLE‐induced diminished distance traveled, as BLE + AYA animals markedly differed from BLE animals (Hedges' *g* = 1.23, 95% CI: 0.28–2.18), while not differing from CTL (Hedges' *g* = 0.12, 95% CI: −0.84 to 1.07).

When evaluating the time spent in the EPM center or number of closed arm entries, no significant differences were detected among groups (*p* > 0.05; Figure 5C,D), demonstrating that these behavioral parameters were not significantly influenced by the experimental conditions.

### 3.4 Effects of ayahuasca on anorexigenic hypothalamic gene expression

The expression of the anorexigenic neuropeptides POMC (pro‐opiomelanocortin) and CART (Cocaine‐ and amphetamine‐regulated transcript) was evaluated in the hypothalamus following the binge‐like eating protocol and ayahuasca treatment, once these genes are strongly associated and modulate food intake.

No significant differences were observed in POMC expression among groups (Figure 6A). The binge‐like eating protocol did not alter POMC expression compared with CTL animals (0.62 ± 0.17 and 0.85 ± 0.23, respectively), and ayahuasca treatment did not significantly modify this parameter despite numerically higher values in the BLE + AYA group (1.37 ± 0.71 versus 0.62 ± 0.17). Effect size estimates were small to moderate, and their 95% confidence intervals included zero (CTL vs. BLE: Hedges' *g* = 0.45, 95% CI: −0.81 to 1.71; BLE vs. BLE + AYA: Hedges' *g* = 0.51, 95% CI: −0.70 to 1.72). These findings suggest that neither the binge‐like eating protocol nor acute ayahuasca administration substantially affected hypothalamic POMC expression under the present experimental conditions.

**FIGURE 6:**
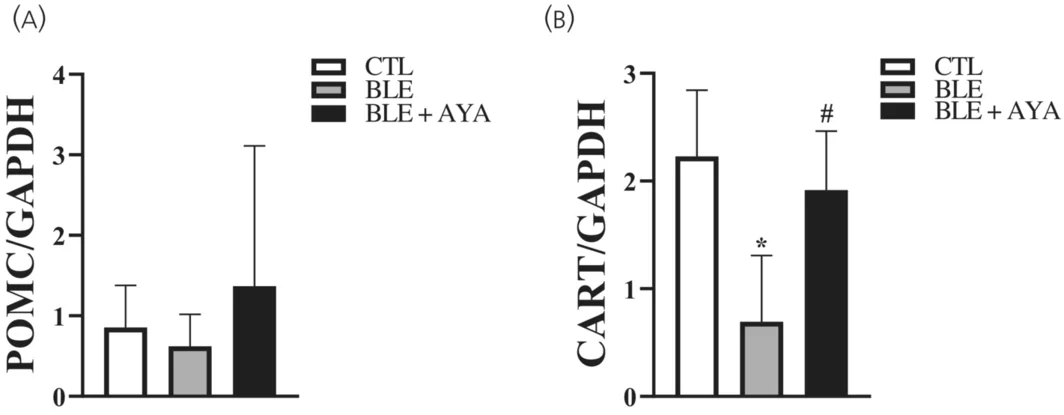
Effects of the binge‐like eating (BLE) protocol and acute ayahuasca administration on hypothalamic POMC and CART mRNA expression. Relative mRNA expression of POMC/GAPDH (A) and CART/GAPDH (B) in the hypothalamus. Data are expressed as mean ± SEM (*n* = 6 per group). *p < 0.05; versus CTL; #*p* < 0.05 versus BLE.

In contrast, CART expression was significantly altered by both the binge‐like eating protocol and ayahuasca treatment (Figure 6B). BLE animals exhibited reduced CART expression compared with CTL animals (0.69 ± 0.30 versus 2.28 ± 0.27), corresponding to a large effect size (Hedges' *g* = 2.22, 95% CI: 0.46 to 3.97). Ayahuasca treatment increased CART expression in BLE animals (1.91 ± 0.24), resulting in a substantial difference relative to the BLE group (Hedges' *g* = 1.88, 95% CI: 0.24 to 3.53). Furthermore, CART expression in BLE + AYA animals was comparable to CTL levels (Hedges' *g* = 0.48, 95% CI: −0.78 to 1.75). These results suggest that the binge‐like eating protocol was associated with reduced hypothalamic CART expression, whereas ayahuasca treatment restored CART expression toward control levels.

## 4 DISCUSSION

In the present study, we demonstrate that the binge‐like eating model is characterized by increased total food intake and sucrose consumption. This phenotype is induced by an intermittent fasting protocol combined with a restricted 4‐h daily access to standard chow and sucrose and is accompanied by anxiogenic‐like behavior and a reduction in hypothalamic CART gene expression. Notably, ayahuasca treatment partially attenuated food and sucrose hyperconsumption, alleviated anxiety‐like behavior, and modulated hypothalamic gene expression profiles.

The intermittent fasting–refeeding protocol employed here successfully reproduced key behavioral hallmarks of binge‐like eating, including increased caloric intake within a restricted temporal window and an enhanced preference for sucrose‐rich solutions. Similar paradigms have been extensively validated as robust preclinical models of binge‐like eating, particularly because they dissociate compulsive intake from homeostatic energy requirements.^36^, ^39^, ^40^, ^41^ Although binge eating disorder is a clinical diagnosis restricted to humans, these animal models capture core neurobehavioral features of the disorder, including heightened reward sensitivity, impaired inhibitory control, and stress‐related behavioral alterations.

Besides that, to enhance the translational validity of the model, adolescent animals were selected, as adolescence represents a critical period for the onset of mental illnesses, including eating disorders.^42^, ^43^ Furthermore, the incomplete maturation of the prefrontal cortex and reward circuitry leads to reduced cognitive control and a higher propensity for compulsive behaviors, making adolescent mice an ideal model for binge‐like behavior.^44^, ^45^, ^46^, ^47^

Fasting per se exerts a significant influence on food reward processing. During fasting conditions, increased orexigenic signaling, particularly via ghrelin, activates central hunger‐related circuits. Ghrelin enhances food motivation by acting on regions such as the ventral tegmental area (VTA), modulating dopaminergic responses to food and promoting the consumption of highly palatable items.^48^ When nutrients such as fats and sugars are consumed during binge episodes, they induce exaggerated dopamine release, which can reinforce compensatory behaviors analogous to those observed in substance abuse.^49^ Consistent with this framework, our results show that fasting periods combined with sucrose availability induce marked alterations in feeding behavior, characterized by increased food and sucrose intake over a short time window. These effects are likely mediated, at least in part, by the interaction between orexigenic signaling and mesolimbic dopamine homeostasis.

Despite the increased food and sucrose consumption observed during refeeding, mice subjected to the binge‐like eating protocol exhibited a reduction in total body weight. This apparent paradox has been consistently reported in intermittent fasting models and likely reflects a sustained negative energy balance that is not fully compensated by episodic hyperphagia.^50^ Prolonged fasting and caloric restriction induce metabolic adaptations that reduce energy expenditure; however, compensatory overeating during refeeding is often insufficient to restore energy balance, resulting in net weight loss.^50^ Thus, the reduction in body weight observed here does not contradict the binge‐like eating phenotype but underscores the dissociation between compulsive intake and long‐term energy homeostasis.

Intermittent fasting paradigms induce pronounced neuroendocrine adaptations that favor orexigenic signaling. Fasting reduces circulating leptin levels while increasing ghrelin secretion, leading to activation of hypothalamic orexigenic neurons and suppression of anorexigenic pathways.^51^, ^52^, ^53^ Upon refeeding, these homeostatic signals converge with sensitized mesolimbic reward circuits, markedly enhancing the motivational salience of palatable foods, particularly rapidly absorbable carbohydrates such as sucrose.^53^

Accordingly, the marked increase in sucrose intake observed in BLE mice may be partially explained by neuroplastic adaptations within dopaminergic and opioid systems. Repeated binge‐like sugar consumption induces persistent dopamine release in the nucleus accumbens, disrupts acetylcholine‐mediated satiety signaling, and downregulates dopamine D2 receptor expression, neuroadaptations that closely resemble those observed in substance use disorders.^54^, ^55^, ^56^, ^57^, ^58^ Additionally, intermittent access to sugar can induce endogenous opioid dependence, reinforcing compulsive intake through negative reinforcement mechanisms.^57^ Together, these findings support the conceptualization of binge eating as an addiction‐like behavioral state driven by maladaptive reward learning.

Using a previously validated dose of ayahuasca (1.76 mg/kg),^37^ as reported in earlier experimental studies, one of the most relevant findings of this study is that a single administration significantly reduced food and sucrose intake during the first hour of refeeding, without affecting intake at later time points. Interestingly, the significant group X time interaction indicates that the effect of ayahuasca was temporally restricted to the initial phase of refeeding rather than reflecting a generalized reduction in food consumption.

Mechanistically, this influence on the motivational process can be attributed to the action of ayahuasca components on 5‐HT receptors.^58^ Furthermore, psilocybin, a psychedelic structurally similar to DMT, has been shown to reduce opioid conditioning and withdrawal through the activation of cortical neurons projecting to the nucleus accumbens (NAc) and to induce dendritic plasticity in this region, both mediated by 5‐HT_2A_ receptors.^62^ Additionally, in a previous study, ayahuasca attenuated the expression of 5‐HT_1A_ and dynorphin in the hippocampus, while also decreasing the dynorphin/prodynorphin ratio in the striatum of mice, which are relevant markers for alcohol dependence.^59^

Coupled with this, ayahuasca and its alkaloids, such as harmine, tetrahydroharmine, and harmaline, have been shown to reduce cocaine and morphine consumption in rats and modulate dopaminergic signaling in the NAc.^30^, ^60^ For instance, the harmine present in ayahuasca increases dopamine efflux into the NAc. Thus, the greater availability of dopamine in this region may have contributed to the reduction of the withdrawal state.^60^ In summary, these findings emphasize the possible involvement of the reward system in our results—particularly the opioid system, which has been shown to be closely linked to the reinforcing effects of hyperpalatable foods,^61^ as well as to the anxiety associated with substance withdrawal.^57^, ^62^

This aligns with another relevant finding of our study: ayahuasca also reversed anxiogenic behaviors in mice experiencing sucrose withdrawal under the BLE protocol, as evidenced by the increased time spent in the open arms of the elevated plus‐maze (EPM). The binge‐like eating protocol here employed is consistent with previous findings demonstrating that withdrawal from highly palatable foods induces anxiety‐like behaviors.^63^ Our results indicate that ayahuasca attenuates this anxiogenic state, supporting previous evidence of its anxiolytic and antidepressant properties in both humans and animal models.^63^, ^64^, ^65^, ^66^ Nevertheless, the literature remains complex, as other studies suggest that ayahuasca may not significantly alter anxiety‐related behaviors^67^, ^68^, ^69^, ^70^ or may even induce anxiogenic effects.^66^, ^69^, ^70^ These contrasting results are likely attributable to methodological differences, including the behavioral assessment timeframe (acute versus 24‐h post‐treatment), ayahuasca alkaloid profiles, and species‐specific responses (e.g., *zebrafish* vs. murine models).

Additionally, the single‐dose treatment paradigm applied in the present study was selected to evaluate the acute and post‐acute therapeutic potential of Ayahuasca. The dose used in this study (1.76 mg DM/Kg) had previously demonstrated an anxiolytic effect in alcohol withdrawal, albeit from repeated administrations.^37^ Thus, this approach was fundamental for the translation of results from clinical trials demonstrating that a single dose of the brew produces rapid and significant antidepressant and anxiolytic effects in humans.^71^, ^72^

Ayahuasca reversal of BLE‐induced anxiogenic‐like behavior observed in this study may be related to serotonergic modulation and to harmine pro‐neurogenic properties, which stimulate neural progenitor cell proliferation and reduce amygdala hyperactivity, therefore contributing to hippocampal circuits restoration once compromised by compulsive eating.^73^, ^74^ Additionally, a previous study demonstrated that ayahuasca increases the release of the inhibitory neurotransmitters GABA and glycine in the amygdala, while reducing it in the hippocampus^75^ This dynamic promotes lower amygdala reactivity and higher hippocampal activity, which may translate into a reduction in anxiety‐like behavior, given the importance of these regions for emotional reactivity and the spatial‐exploratory processing assessed in the EPM.^76^, ^77^

This interpretation is consistent with clinical and preclinical evidence indicating that ayahuasca exerts beneficial effects on addictive behaviors and maladaptive reward processing.^63^, ^78^, ^79^ In clinical contexts, ayahuasca administration has been associated with reductions in alcohol and cocaine use, improvements in psychological well‐being, and sustained changes in impulse control and emotional regulation.^33^, ^34^, ^64^ Importantly, these effects often emerge rapidly after a single or few administrations, suggesting a profound but transient modulation of neural circuits governing motivation and behavior. The absence of persistent effects on food intake beyond the day of administration in our study further supports the notion that ayahuasca primarily influences acute motivational drive rather than long‐term metabolic regulation.

In addition to modulating binge‐like eating behavior, ayahuasca also reversed the anxiogenic‐like behavior induced by the binge‐like eating protocol, as evidenced by increased time spent in the open arms of the elevated plus maze (EPM). The binge‐like eating protocol employed here is consistent with previous findings demonstrating that withdrawal from highly palatable foods induces anxiety‐like behaviors.^65^ Our results indicate that ayahuasca attenuates this anxiogenic state, in line with studies reporting anxiolytic effects of the brew.^66^, ^67^, ^68^ Nevertheless, these findings remain controversial, as other studies suggest that ayahuasca, depending on dose and experimental context, may not significantly alter anxiety‐related behaviors.^69^, ^70^, ^73^, ^74^

Chronic exposure to intermittent fasting combined with binge‐like eating has been described as a potent activator of stress‐related pathways, including the hypothalamic–pituitary–adrenal (HPA) axis, which may further exacerbate compulsive eating through interactions with reward circuits.^39^ Furthermore, the daily short‐term social isolation (4 h) required for individual food measurement likely acted as an acute ethological stressor that synergized with these dietary variables to potentiate the binge‐like and anxiogenic phenotypes, a dynamic properly controlled in our study, as baseline animals were subjected to the exact same isolation paradigm.^80^, ^81^ Given the persistent activation of stress systems and the resulting emotional dysregulation induced by this protocol, elucidating the neurobiological mechanisms through which ayahuasca attenuates both anxiety and behavioral impairments associated with compulsive eating is particularly relevant. In this regard, the anxiolytic‐like effects observed here are consistent with evidence demonstrating antidepressant and anxiolytic properties of ayahuasca in both humans and animal models.^28^, ^29^, ^63^

Ayahuasca is a pan‐Amazonian psychoactive brew prepared from the decoction of *Banisteriopsis caapi*, rich in β‐carbolines such as harmine, tetrahydroharmine, and harmaline, combined with the leaves of *Psychotria viridis*, a source of N,N‐dimethyltryptamine (DMT).^82^ β‐carbolines, particularly harmine, inhibit monoamine oxidase A (MAO‐A), enabling the oral bioavailability of DMT, which acts as an agonist at serotonergic 5‐HT₁A/2A/2C receptors in frontal and paralimbic regions.^83^ Accordingly, the reversal of anxiogenic‐like behavior observed in this study may be related to serotonergic modulation and to the pro‐neurogenic properties of harmine, which stimulates neural progenitor cell proliferation and reduces amygdala hyperactivity, thereby contributing to the restoration of hippocampal circuits compromised by compulsive eating.^84^, ^85^

In addition to its anxiolytic‐like effects, BLE mice exhibited a significant reduction in total distance travel, which was reversed by ayahuasca treatment. Although these findings contrast with studies reporting reduced or unchanged locomotor activity following ayahuasca administration in the open field,^67^, ^68^, ^70^, ^73^, ^86^ no evidence of generalized locomotor suppression was observed in our analyses. Reduced closed‐arm entries in the EPM have been proposed as an index of diminished exploratory activity, an effect reported elsewhere^66^ but not detected in the present study. These discrepancies may reflect differences in dose, timing, or behavioral paradigms. Notably, ayahuasca has been shown to promote structural plasticity, and increased synaptic connectivity and neural circuit reorganization may contribute to the restoration of environmental responsiveness and exploratory motivation, thereby attenuating apathy associated with the binge‐like eating phenotype.^83^, ^85^

In parallel with adaptations in corticolimbic and mesolimbic circuits, the regulation of feeding behavior also involves central homeostatic mechanisms mediated by hypothalamic nuclei. The arcuate nucleus expresses anorexigenic genes such as POMC and CART, which reduce food intake and increase energy expenditure in response to nutrient and neuroendocrine signals.^87^ Although no differences in POMC expression were observed between CTL and BLE groups, the binge‐like eating protocol induced a significant reduction in CART expression in BLE mice. Reductions in CART expression have been reported in conditions associated with decreased fat mass and leptin levels,^88^ as well as in animal models of food restriction with varying durations and severities.^89^, ^90^ These findings provide a plausible mechanistic explanation for the reduced CART expression observed in our model, likely reflecting fasting‐induced attenuation of hypothalamic satiety signaling.

Ayahuasca treatment increased CART expression relative to BLE mice, an effect that may be associated with the observed reductions in food and sucrose intake in the BLE + AYA group. DMT is a potent activator of serotonergic receptors, including 5‐HT_2_A and 5‐HT_2_C, which are expressed in both the central nervous system and the hypothalamus.^91^ DMT administration in male and female Sprague–Dawley rats reduces food intake without altering locomotor activity or energy expenditure parameters, such as respiratory exchange rate (RER) or heat production,^92^ suggesting a selective modulation of hypothalamic circuits involved in feeding and satiety.

Serotonin plays a central role in the regulation of food intake by integrating homeostatic and hedonic circuits.^93^ Within both systems, serotonergic signaling contributes to metabolic regulation by suppressing food intake and attenuating reward‐driven, motivational feeding.^93^ Given its pharmacological profile, ayahuasca, and particularly its DMT component, acts on serotonergic pathways,^91^ which may underline the reduced food and sucrose intake observed in BLE + AYA mice. Importantly, these effects are consistent with modulation of hypothalamic CART signaling rather than broad suppression of feeding behavior.

Despite these promising findings, several limitations of the present study should be acknowledged. First, the exclusive use of male mice does not allow us to address potential sex‐dependent differences in the effects of both the BLE protocol and ayahuasca, thereby limiting the generalizability of our results to females. Second, the absence of a control group treated with ayahuasca in the absence of the BLE protocol and the use of a single ayahuasca dose constrains the interpretation of ayahuasca's effects independently of binge‐like eating induction, although previous studies have specifically addressed the behavioral and neurobiological consequences of ayahuasca administration in non–binge‐eating conditions. Furthermore, anxiety‐like behavior was evaluated exclusively using the Elevated Plus Maze (EPM). While the EPM is a well‐established and validated model for assessing anxiety‐related behavior in rodents, the use of additional behavioral paradigms would have strengthened the behavioral characterization and increased confidence in the interpretation of the anxiolytic‐like effects observed following ayahuasca administration. Finally, although the alkaloid content of the ayahuasca preparation was analytically characterized, ayahuasca remains a complex botanical formulation whose pharmacological effects result from the interaction of multiple bioactive compounds. Consequently, variations in the relative concentrations of DMT and β‐carbolines may affect biological outcomes, limiting the identification of a single optimal dose applicable to all preparations.

## 5 CONCLUSIONS

In summary, our findings demonstrate that intermittent fasting combined with restricted access to palatable foods induces a robust binge‐like eating phenotype in mice, accompanied by anxiogenic‐like behavior and alterations in hypothalamic CART expression. A single administration of ayahuasca selectively attenuated the initial binge‐like eating response and reversed anxiety‐related behavioral alterations, without inducing nonspecific suppression of food intake or sustained effects on energy consumption.

These results suggest that ayahuasca primarily modulates the motivational and emotional components of binge‐like eating, likely through interactions with reward‐related and stress‐responsive circuits rather than through direct effects on long‐term homeostatic feeding regulation. Collectively, this study provides experimental evidence supporting the relevance of ayahuasca as a tool for investigating neurobiological mechanisms underlying compulsive eating and highlights its potential translational value for the development of novel psychedelic‐based interventions for binge eating disorder.

## AUTHOR CONTRIBUTIONS

**Cristian Ferreira Corona:** Conceptualization; methodology; data curation; writing – original draft; formal analysis. **Felipe Alves Alencar Lima:** Conceptualization; methodology; data curation; writing – original draft. **Jessica Sabrina Canedo da Silva Sumensse:** Methodology; data curation; supervision; validation. **Kevin Donansan Carvalho:** Methodology; data curation; writing – original draft; conceptualization. **Pedro Henrique Grignet:** Methodology; validation; visualization; data curation. **Heloisa Deola Confortim:** Conceptualization; writing – original draft; writing – review and editing; project administration. **Maria Claudia Gross:** Conceptualization; writing – original draft; writing – review and editing; project administration. **Fabiana Aidar Firmino:** Conceptualization; writing – original draft; writing – review and editing; methodology. **Fernanda Ferreira Evangelista:** Conceptualization; writing – original draft; writing – review and editing; methodology; data curation; formal analysis. **Francisney Pinto Nascimento:** Conceptualization; methodology; writing – review and editing; writing – original draft. **Jean Franciesco Vettorazzi:** Conceptualization; funding acquisition; writing – original draft; writing – review and editing; validation; methodology; formal analysis; data curation; supervision.

## FUNDING INFORMATION

This work was supported by PROAP (Program to Support Graduate Studies) from CAPES—Coordination for the Improvement of Higher Education Personnel and Araucária Foundation for the Support of Scientific and Technological Development in the State of Paraná.

## CONFLICT OF INTEREST STATEMENT

The authors declare no conflicts of interest.

## ETHICS STATEMENT

All experimental procedures involving animals were approved by the Ethics Committee for the Use of Animals (CEUA) of the Federal University of Latin American Integration (UNILA) and were conducted in accordance with national and international guidelines for the care and use of laboratory animals.

## Supporting information


**TABLE S1.** Two‐way repeated‐measures ANOVA and Bonferroni‐corrected post hoc comparisons for body weight during the 10‐day acquisition phase (Figure 1A).
**TABLE S2.** Two‐way repeated‐measures anova and bonferroni‐corrected post hoc comparisons for 1 h standard chow intake during the 10‐day acquisition phase (Figure 1C).
**TABLE S3.** Two‐way repeated‐measures anova and bonferroni‐corrected post hoc comparisons for cumulative 4 h standard chow intake during the 10‐day acquisition phase (Figure 1D).
**TABLE S4.** Bonferroni‐corrected post hoc comparisons for 1 h sucrose intake during the 10‐day acquisition phase (Figure 2A).
**TABLE S5.** Bonferroni‐corrected post hoc comparisons for cumulative 4 h sucrose intake during the 10‐day acquisition phase (Figure 2A).
**TABLE S6.** Two‐way repeated‐measures anova and bonferroni‐corrected post hoc comparisons for 1 h and cumulative 4 h standard chow intake following acute ayahuasca administration (Figure 3A,C).
**TABLE S7A.** Two‐way repeated‐measures ANOVA for 1 h sucrose intake (Figure 4A).
**TABLE S7B.** Two‐way repeated‐measures ANOVA for 4 h sucrose intake (Figure 4C).
**TABLE S8A.** Bonferroni‐corrected post hoc comparisons for 1 h sucrose intake (Figure 4A).
**TABLE S8B.** Bonferroni‐corrected post hoc comparisons for 4 h sucrose intake (Figure 4C).

## Data Availability

The data that support the findings of this study are available from the corresponding author upon reasonable request.
