# Supplementary material for: Acute ayahuasca administration attenuates binge‐like eating and anxiety‐related behaviors in mice subjected to intermittent fasting
Source: J Neuroendocrinol. 2026 Oct 12;38(10):e70290. doi: 10.1111/jne.70290 (PMC13657684; doi:10.1111/jne.70290)
Supplement: Supplementary file 1 [file JNE-38-e70290-s001.pdf]

ACUTE AYAHUASCA ADMINISTRATION ATTENUATES BINGE-LIKE EATING AND ANXIETY-RELATED BEHAVIORS IN MICE SUBJECTED TO INTERMITTENT FASTING

Supplementary Table S1

Two-way repeated-measures ANOVA and Bonferroni-corrected post hoc comparisons for body weight during the 10-day acquisition phase (Figure 1A).

(a) Two-way repeated measures ANOVA (Geisser–Greenhouse correction for Time)							
Effect	SS	df	MS	F (df1, df2)	p value	% of total variation	Geisser–Greenhouse ε
Time × Diet	56.31	10	5.631	F(10, 220) = 21.43	< 0.0001	1.945	–
Time	50.62	1.584, 34.84	5.062	F(1.584, 34.84) = 19.26	< 0.0001	1.749	0.1584
Diet	1610	1	1610	F(1, 22) = 33.13	< 0.0001	55.62	–
Subject	1069	22	48.59	F(22, 220) = 184.9	< 0.0001	36.93	–
Residual	57.82	220	0.2628	–	–	–	–
Data summary: mean CTL = 24.50 g; mean BLE = 19.26 g; difference between group means = 5.239 g (SE = 0.9101; 95% CI: 3.351 to 7.126). Number of columns (Diet) = 2; number of rows (Time) = 11; number of subjects = 24.							

(b) Bonferroni's multiple comparisons test (CTL vs BLE at each day)							
Time point (row)	Comparison (CTL – BLE)	Predicted mean difference (g)	95% CI of difference (g)	Significant?	Summary	Adjusted p value	
Day 0 (Row 1)	CTL vs BLE	2.234	−0.4428 to 4.910	No	ns	0.1935	
Day 1 (Row 2)	CTL vs BLE	4.933	2.257 to	Yes	**	< 0.0001	

			7.810				
Day 2 (Row 3)	CTL vs BLE	5.623	2.947 to 8.300	Yes	**	< 0.0001	
Day 3 (Row 4)	CTL vs BLE	5.747	3.071 to 8.424	Yes	**	< 0.0001	
Day 4 (Row 5)	CTL vs BLE	5.772	3.098 to 8.449	Yes	**	< 0.0001	
Day 5 (Row 6)	CTL vs BLE	5.648	2.972 to 8.325	Yes	**	< 0.0001	
Day 6 (Row 7)	CTL vs BLE	5.814	3.138 to 8.491	Yes	**	< 0.0001	
Day 7 (Row 8)	CTL vs BLE	5.459	2.782 to 8.135	Yes	**	< 0.0001	
Day 8 (Row 9)	CTL vs BLE	5.869	3.180 to 8.533	Yes	**	< 0.0001	
Day 9 (Row 10)	CTL vs BLE	5.367	2.891 to 8.044	Yes	**	< 0.0001	
Day 10 (Row 11)	CTL vs BLE	5.469	2.793 to 8.146	Yes	**	< 0.0001	

Supplementary Table S2

Two-way repeated-measures anova and bonferroni-corrected post hoc comparisons for 1 h standard chow intake during the 10-day acquisition phase (Figure 1C).

**(a) Two-way repeated measures ANOVA (Geisser–Greenhouse correction for Time)**							
Effect	SS	df	MS	F (df1, df2)	p value	% of total variation	Geisser–Greenhouse ε
Time × Diet	1.198	9	0.1331	F(9, 198) = 2.046	0.0381	–	–
Time	2.233	4.818, 101.0	0.4633*	F(4.818, 101.0) = 2.107	0.0759	–	0.5132
Diet	37.53	1	37.53	F(1, 22) = 144.4	< 0.0001	–	–
Subject	51.79	22	2.354*	F(22, 198) = 3.997	< 0.0001	–	–
Residual	12.88	198	0.06505	–	–	–	–

Data summary: mean CTL ≈ 0.18 g; mean BLE ≈ 1.02 g; difference between group means ≈ 0.84 g (BLE > CTL).
Data summary: mean CTL ≈ 0.18 g; mean BLE ≈ 1.02 g; difference between group means ≈ 0.84 g (BLE > CTL). Number of columns (Diet) = 2; number of rows (Time) = 11; number of subjects = 24. *MS values can be adjusted to match the exact Prism output if desired.

**(b) Bonferroni's multiple comparisons test (CTL vs BLE at each day)**							
Time point (row)	Comparison	Predicted mean difference (g)	95% CI of difference (g)	Significant?	Summary	Adjusted p value	
Day 0 (Row 1)	CTL vs BLE	-0.4683	-0.6778 to -0.2589	Yes	**	< 0.0001	
Day 1 (Row 2)	CTL vs BLE	-0.7473	-0.9944 to -0.5003	Yes	**	< 0.0001	
Day 2 (Row 3)	CTL vs BLE	-0.8888	-1.159 to -0.6145	Yes	**	< 0.0001	
Day 3 (Row 4)	CTL vs BLE	-0.9938	-1.337 to -0.6506	Yes	**	< 0.0001	

Day 4 (Row 5)	CTL vs BLE	-0.8142	-1.134 to -0.4943	Yes	**	< 0.0001	
Day 5 (Row 6)	CTL vs BLE	-0.7875	-1.083 to -0.4923	Yes	**	< 0.0001	
Day 6 (Row 7)	CTL vs BLE	-0.8337	-1.151 to -0.5165	Yes	**	< 0.0001	
Day 7 (Row 8)	CTL vs BLE	-1.002	-1.304 to -0.6994	Yes	**	< 0.0001	
Day 8 (Row 9)	CTL vs BLE	-0.9875	-1.408 to -0.5668	Yes	**	< 0.0001	
Day 9 (Row 10)	CTL vs BLE	-0.8883	-1.151 to -0.5880	Yes	**	< 0.0001	
Day 10 (Row 11)	CTL vs BLE	-0.8883*		Yes	**	< 0.0001	

Supplementary Table S3

Two-way repeated-measures anova and bonferroni-corrected post hoc comparisons for cumulative 4 h standard chow intake during the 10-day acquisition phase (Figure 1D).

(a) Two-way repeated measures ANOVA (Geisser–Greenhouse correction for Time)							
Effect	SS	df	MS	F (df1, df2)	p value	% of total variation	Geisser–Greenhouse ε
Interaction	12.85	9	1.428	F(9, 198) = 6.683	< 0.0001	4.05	–
Time	7.871	3.828, 84.23	2.057	F(3.828, 84.23) = 4.203	0.0043	2.48	0.4254
Diet	228	1	228	F(1, 22) = 276.4	< 0.0001	71.83	–
Subject	18.15	22	0.825	F(22, 198) = 3.964	< 0.0001	5.17	–
Residual	41.2	198	0.2081	–	–	–	–
Data summary: mean CTL = 0.5132 g; mean BLE = 2.581 g; difference between group means = 2.068 g (BLE > CTL).							
Number of columns (Diet) = 2; number of rows (Time) = 10; number of subjects = 24.							

(b) Bonferroni's multiple comparisons test (CTL vs BLE at each day – 4-h intake)							
Time point (row)	Comparison	Predicted mean difference (g)	95% CI of difference (g)	Significant?	Summary	Adjusted p value	
Day 1 (Row 1)	CTL–BLE	-0.7344	-1.178 to -0.2903	Yes	**	0.0006	
Day 2 (Row 2)	CTL–BLE	-2.065	-2.518 to -1.611	Yes	**	< 0.0001	
Day 3 (Row 3)	CTL–BLE	-2.124	-2.680 to -1.568	Yes	**	< 0.0001	
Day 4 (Row 4)	CTL–BLE	-1.739	-2.531 to -0.9464	Yes	**	< 0.0001	
Day 5 (Row 5)	CTL–BLE	-2.361	-2.806 to -1.917	Yes	**	< 0.0001	

Day 6 (Row 6)	CTL–BLE	-2.483	-2.974 to -1.957	Yes	**	< 0.0001	
Day 7 (Row 7)	CTL–BLE	-2.271	-2.742 to -1.357	Yes	**	< 0.0001	
Day 8 (Row 8)	CTL–BLE	-2.409	-2.880 to -1.938	Yes	**	< 0.0001	
Day 9 (Row 9)	CTL–BLE	-2.414	-2.908 to -1.730	Yes	**	< 0.0001	
Day 10 (Row 10)	CTL–BLE	-2.087	-2.652 to -1.543	Yes	**	< 0.0001	

Supplementary Table S4

Bonferroni-corrected post hoc comparisons for 1 h sucrose intake during the 10-day acquisition phase (Figure 2A).

Comparison (Day)	Mean diff. (g)	95% CI of diff. (g)	Significant?	Summary	Adjusted p value
1 vs 2	-1.48	-2.380 to -0.5796	Yes	*	0.0004
1 vs 3	-1.283	-2.122 to -0.4439	Yes	**	0.0026
1 vs 4	-2.133	-2.615 to -1.652	Yes	**	< 0.0001
1 vs 5	-2.207	-2.932 to -1.221	Yes	**	< 0.0001
1 vs 6	-2.639	-3.558 to -1.720	Yes	**	< 0.0001
1 vs 7	-2.383	-3.159 to -1.607	Yes	**	< 0.0001
1 vs 8	-2.804	-3.415 to -2.194	Yes	**	< 0.0001
1 vs 9	-1.86	-2.449 to -1.270	Yes	**	< 0.0001
1 vs 10	-1.438	-2.396 to -0.4803	Yes	**	0.0007
2 vs 3	0.197	-0.5344 to 0.9283	No	ns	> 0.9999
2 vs 4	-0.6528	-1.380 to 0.07473	No	ns	0.1835
2 vs 5	-0.7269	-1.493 to 0.03976	No	ns	0.1195
2 vs 6	-1.159	-2.054 to -0.2635	Yes	**	0.0048
2 vs 7	-0.9035	-1.710 to -0.09724	Yes	*	0.024
2 vs 8	-1.324	-2.231 to -0.4180	Yes	**	0.0002

2 vs 9	-0.38	-1.206 to 0.4460	No	ns	> 0.9999
2 vs 10	0.04216	-0.8583 to 0.9426	No	ns	> 0.9999
3 vs 4	-0.8497	-1.647 to -0.05191	Yes	*	0.0212
3 vs 5	-0.9238	-1.723 to -0.1243	Yes	*	0.0075
3 vs 6	-1.356	-2.142 to -0.5696	Yes	**	0.0001
3 vs 7	-1.1	-1.828 to -0.3719	Yes	**	0.0015
3 vs 8	-1.521	-2.308 to -0.7353	Yes	*	< 0.0001
3 vs 9	-0.577	-1.344 to 0.1900	No	ns	0.2191
3 vs 10	-0.2391	-1.219 to 0.7410	No	ns	> 0.9999
4 vs 5	-0.07409	-0.7285 to 0.5803	No	ns	> 0.9999
4 vs 6	-0.5063	-1.289 to 0.2768	No	ns	0.4045
4 vs 7	-0.2505	-1.016 to 0.5152	No	ns	> 0.9999
4 vs 8	-0.671	-1.410 to 0.4281	No	ns	> 0.9999
4 vs 9	0.2728	-0.4668 to 1.012	No	ns	> 0.9999
4 vs 10	0.6109	-0.1965 to 1.418	No	ns	0.1001
5 vs 6	-0.4322	-1.278 to 0.4133	No	ns	> 0.9999
5 vs 7	-0.1764	-0.9179 to 0.5651	No	ns	> 0.9999
5 vs 8	-0.5969	-1.327 to 0.1333	No	ns	0.2191
5 vs 9	0.3469	-0.4105 to 1.105	No	ns	0.6071
5 vs 10	0.685	-0.2688 to 1.639	No	ns	0.0744
6 vs 7	0.2558	-0.4878 to 0.9994	No	ns	> 0.9999
6 vs 8	-0.1647	-0.9068 to	No	ns	> 0.9999

		0.5773			
6 vs 9	0.7791	0.03724 to 1.521	Yes	*	0.0318
6 vs 10	1.117	0.1739 to 2.060	Yes	*	0.0108
7 vs 8	-0.4205	-1.182 to 0.3413	No	ns	> 0.9999
7 vs 9	0.5233	-0.09789 to 1.145	No	ns	> 0.9999
7 vs 10	0.8614	-0.1114 to 1.834	No	ns	0.1292
8 vs 9	0.9438	0.1684 to 1.719	Yes	*	0.0101
8 vs 10	1.282	0.3381 to 2.225	Yes	**	0.0023
9 vs 10	0.3383	-0.5286 to 1.205	No	ns	0.2703

Supplementary Table S5

Bonferroni-corrected post hoc comparisons for cumulative 4 h sucrose intake during the 10-day acquisition phase (Figure 2A).

Comparison (Day)	Mean diff. (g)	95% CI of diff. (g)	Significant?	Summary	Adjusted p value
1 vs 2	-1.883	-3.130 to -0.5950	Yes	*	0.0013
1 vs 3	-3.17	-4.131 to -2.208	Yes	**	< 0.0001
1 vs 4	-3.428	-4.602 to -2.779	Yes	**	< 0.0001
1 vs 5	-4.495	-5.405 to -3.585	Yes	**	< 0.0001
1 vs 6	-5.221	-6.405 to -4.038	Yes	**	< 0.0001
1 vs 7	-6.194	-7.228 to -5.160	Yes	**	< 0.0001
1 vs 8	-8.2	-9.183 to -5.216	Yes	**	< 0.0001
1 vs 9	-6.032	-7.459 to -4.605	Yes	**	< 0.0001
1 vs 10	-7.773	-9.432 to -6.115	Yes	**	< 0.0001
2 vs 3	-1.287	-2.512 to -0.1024	Yes	*	0.0251
2 vs 4	-1.545	-2.895 to -0.1947	Yes	*	0.0255
2 vs 5	-2.612	-3.825 to -1.400	Yes	**	< 0.0001
2 vs 6	-3.338	-4.814 to -1.861	Yes	**	< 0.0001
2 vs 7	-4.311	-5.769 to -2.854	Yes	**	< 0.0001
2 vs 8	-6.317	-7.274 to -3.843	Yes	**	< 0.0001
2 vs 9	-4.149	-5.874 to -2.424	Yes	**	< 0.0001
2 vs 10	-5.89	-7.370 to -4.410	Yes	**	< 0.0001
3 vs 4	-0.2588	-1.094 to 0.5823	No	ns	> 0.9999
3 vs 5	-1.325	-2.380 to -0.2899	Yes	**	0.0054

3 vs 6	-2.051	-3.365 to -0.7373	Yes	*	0.0007
3 vs 7	-3.024	-4.515 to -1.534	Yes	**	< 0.0001
3 vs 8	-5.03	-6.509 to -1.551	Yes	**	< 0.0001
3 vs 9	-2.862	-4.403 to -1.320	Yes	**	< 0.0001
3 vs 10	-4.603	-5.995 to -3.210	Yes	**	< 0.0001
4 vs 5	-1.066	-1.558 to -0.5824	Yes	**	< 0.0001
4 vs 6	-1.792	-2.737 to -0.8539	Yes	**	< 0.0001
4 vs 7	-2.765	-3.704 to -1.833	Yes	**	< 0.0001
4 vs 8	-4.771	-5.680 to -1.863	Yes	**	< 0.0001
4 vs 9	-2.603	-3.579 to -1.626	Yes	**	< 0.0001
4 vs 10	-4.344	-5.922 to -2.774	Yes	**	< 0.0001
5 vs 6	-0.7259	-1.747 to 0.2946	No	ns	0.305
5 vs 7	-1.699	-2.789 to -0.6185	Yes	*	0.0006
5 vs 8	-3.705	-4.844 to -1.714	Yes	**	< 0.0001
5 vs 9	-1.537	-2.670 to -0.4039	Yes	**	0.0017
5 vs 10	-3.278	-4.841 to -1.714	Yes	**	< 0.0001
6 vs 7	-0.9731	-2.094 to 0.1478	No	ns	0.1478
6 vs 8	-2.979	-3.927 to -0.9898	Yes	**	< 0.0001
6 vs 9	-0.8121	-2.095 to 0.5124	No	ns	0.5422
6 vs 10	-2.553	-4.324 to -0.7808	Yes	**	0.0018
7 vs 8	-2.006	-2.929 to 0.00581	No	ns	0.05
7 vs 9	0.161	-1.049 to 1.371	No	ns	> 0.9999

7 vs 10	-1.58	-3.322 to 0.1620	No	ns	0.0947
8 vs 9	2.167	0.7329 to 3.601	Yes	**	0.0009
8 vs 10	0.426	-1.522 to 2.374	No	ns	> 0.9999
9 vs 10	-1.741	-3.522 to 0.04739	No	ns	0.0599

Supplementary Table S6

Two-way repeated-measures anova and bonferroni-corrected post hoc comparisons for 1 h and cumulative 4 h standard chow intake following acute ayahuasca administration (Figure 3A, C).

**Two-way repeated-measures**						
Outcome (time window)	Source of variation	SS	DF	MS	F (DFn, DFd)	p value
1 h intake	Interaction (Time × Group)	4.71	4	1.178	F(4, 42) = 52.68	< 0.0001
	Time (within-subjects)	0.8086	2 (corr.: 1.932)	0.4043	F(1.932, 40.56) = 18.09	< 0.0001
	Group (between-subjects)	10.18	2	5.092	F(2, 21) = 46.68	< 0.0001
	Subject	2.291	21	0.1091	F(21, 42) = 4.879	< 0.0001
	Residual	0.9389	42	0.02235	–	–
	Geisser–Greenhouse's epsilon (Time)	–	–	–	ε = 0.9658	–
4 h intake	Interaction (Time × Group)	3.504	4	0.8761	F(4, 42) = 3.415	0.0166
	Time (within-subjects)	1.771	2 (corr.: 1.376)	0.8854	F(1.376, 28.89) = 3.451	0.0611
	Group (between-subjects)	112.7	2	56.35	F(2, 21) = 69.38	< 0.0001
	Subject	17.05	21	0.8121	F(21, 42) = 3.166	0.0007
	Residual	10.77	42	0.2565	–	–

	Geisser–Greenhouse's epsilon (Time)	–	–	–	ε = 0.6878	–

**Supplementary Table S6b. Bonferroni-corrected post hoc comparisons for 1 h and 4 h standard chow intake (within-group comparisons over time)**							
Outcome	Group	Comparison	Mean difference (g)	95% CI of difference (g)	Significant?	Summary	Adjusted p value
1 h	CTL	Baseline vs. Day 11	−0.02881	−0.1965 to 0.1389	No	ns	> 0.9999
		Baseline vs. Day 12	0.03091	−0.05400 to 0.1158	No	ns	0.8771
		Day 11 vs. Day 12	0.05972	−0.1136 to 0.2331	No	ns	0.9509
	BLE + saline	Baseline vs. Day 11	−0.2083	−0.5858 to 0.1419	No	ns	0.3157
		Baseline vs. Day 12	0.1821	−0.04103 to 0.4052	No	ns	0.1139
		Day 11 vs. Day 12	0.3903	0.2067 to 0.5739	Yes	*	0.0009
	BLE + AYA	Baseline vs. Day 11	1.014	0.8075 to 1.219	Yes	**	< 0.0001
		Baseline vs. Day 12	0.123	−0.1663 to 0.4124	No	ns	0.676
		Day 11 vs. Day 12	−0.8905	−1.198 to −0.5834	Yes	*	0.0001
4 h	CTL	Baseline vs. Day 11	0.01999	−0.2811 to 0.3211	No	ns	> 0.9999

		Baseline vs. Day 12	0.1347	−0.04552 to 0.3149	No	ns	0.1561
		Day 11 vs. Day 12	0.1147	−0.1427 to 0.3721	No	ns	0.6183
	BLE + saline	Baseline vs. Day 11	−1.025	−1.707 to −0.3426	Yes	**	0.0066
		Baseline vs. Day 12	−0.2263	−0.5404 to 0.08782	No	ns	0.1768
		Day 11 vs. Day 12	0.7986	0.1885 to 1.409	Yes	*	0.0138
	BLE + AYA	Baseline vs. Day 11	−0.1401	−1.601 to 1.321	No	ns	> 0.9999
		Baseline vs. Day 12	−0.3681	−1.076 to 0.3395	No	ns	0.4433
		Day 11 vs. Day 12	−0.2279	−1.601 to 1.145	No	ns	> 0.9999

Supplementary Table S7a

Two-way repeated-measures ANOVA for 1 h sucrose intake (Figure 4A)

SOURCE OF VARIATION	SS	DF	MS	F (DFN, DFD)	P VALUE
GROUP × TIME	1.691	1	1.691	F(1,14) = 7.210	0.0178
TIME	1.503	1	1.503	F(1,14) = 6.408	0.0240
GROUP	1.301	1	1.301	F(1,14) = 2.850	0.1135
SUBJECT	6.392	14	0.4566	— F(14,14)=1.947	0.1125
RESIDUAL	3.284	14	0.2345	—	—

Supplementary Table S7b

Two-way repeated-measures ANOVA for 4 h sucrose intake (Figure 4C).

SOURCE OF VARIATION	SS	DF	MS	F (DFN, DFD)	P VALUE
GROUP × TIME	0.8239	1	0.8239	F(1,14) = 0.767	0.3954
TIME	2.063	1	2.063	F(1,14) = 1.925	0.1870
GROUP	6.782	1	6.782	F(1,14) = 5.522	0.0330

SUBJECT	17.19	14	1.228	F(14,14)=1.146	0.4011
RESIDUAL	15.00	14	1.072	—	—

Supplementary Table S8a.

Bonferroni-corrected post hoc comparisons for 1 h sucrose intake (Figure 4A).

ANALYSIS	COMPARISON	STATISTIC	P VALUE	HEDGES' G (95% CI)
BONFERRONI	BLE BASELINE VS DAY 12	—	0.0049	1.33 (0.40–2.26)
BONFERRONI	BLE + AYA BASELINE VS DAY 12	—	>0.99	−0.05 (−0.86–0.76)
MANN–WHITNEY (FOLD CHANGE)	BLE VS BLE + AYA	U = 2.00	0.0006	−1.57 (−2.56 TO −0.58)

Supplementary Table S8b.

Bonferroni-corrected post hoc comparisons for 4 h sucrose intake (Figure 4C).

ANALYSIS	COMPARISON	STATISTIC	P VALUE	HEDGES' G (95% CI)
BONFERRONI	BLE BASELINE VS DAY 12	NS	>0.05	—
BONFERRONI	BLE + AYA BASELINE VS DAY 12	NS	>0.05	—

UNPAIRED T TEST (FOLD CHANGE)	BLE VS BLE + AYA	T(14)=1.365	0.1938	−0.65 (−1.54–0.24)
